# Supplementary material for: Chiral excitonic order from twofold van Hove singularities in kagome metals
Source: Nat Commun. 2023 Feb 4;14:605. doi: 10.1038/s41467-023-35987-2 (PMC9899280; doi:10.1038/s41467-023-35987-2)
Supplement: Supplementary file 1 — Supplementary Information [file 41467_2023_35987_MOESM1_ESM.pdf]

SUPPLEMENTARY MATERIAL
"Chiral excitonic order from twofold van Hove singularities in kagome metals"

Harley D. Scammell,[1,2,*] Julian Ingham,[3,†] Tommy Li,[4] and Oleg P. Sushkov[1,2]

[1] *School of Physics, University of New South Wales, Sydney 2052, Australia*

[2] *Australian Research Council Centre of Excellence in Future Low-Energy Electronics Technologies, University of New South Wales, Sydney 2052, Australia*

[3] *Physics Department, Boston University, Commonwealth Avenue, Boston, MA 02215, USA*

[4] *Dahlem Center for Complex Quantum Systems and Fachbereich Physik, Freie Universität Berlin, Arnimallee 14, 14195 Berlin, Germany*

CONTENTS

I. Interactions 2
A. Patch model 2
B. Tight binding model 2
C. Patch Model – initial coupling estimates 3

II. Flow equations for the couplings 6

III. Order parameter gap equations 8

IV. RG fixed rays 9
A. Model: Kagome $\kappa = 2$ 10
B. Model: Kagome $\kappa = 1$ 10
C. Model: Honeycomb $\kappa = 1$ 11

V. Free energy expansion 12

VI. Honeycomb-kagome bilayer toy model 14

VII. Properties of the chiral excitonic condensate 15
A. Honeycomb-kagome bilayer 15
B. Two-orbital kagome systems 17

* h.scammell@unsw.edu.au
† jingham@bu.edu

I. INTERACTIONS

A. Patch model

In the kagome tight-binding model, the wavefunctions at the pure (+) and mixed (−) vHS have the following sublattice structure near the M-points,

$$
\begin{aligned}
|\boldsymbol{M}_1, +\rangle &= \hat{A}, & |\boldsymbol{M}_1, -\rangle &= \frac{1}{\sqrt{2}}\left(\hat{B}+\hat{C}\right), \\
|\boldsymbol{M}_2, +\rangle &= \hat{B}, & |\boldsymbol{M}_2, -\rangle &= \frac{1}{\sqrt{2}}\left(\hat{A}+\hat{C}\right), \\
|\boldsymbol{M}_3, +\rangle &= \hat{C}, & |\boldsymbol{M}_3, -\rangle &= \frac{1}{\sqrt{2}}\left(\hat{A}+\hat{B}\right).
\end{aligned} \tag{S1}
$$

where $\hat{A}, \hat{B}, \hat{C}$ are basis vectors indicating support on the A, B, C sublattices. Due to the different sublattice structure of the wavefunctions for the pure and mixed states, the corresponding interactions evaluated on the pure and mixed vHS have different intraflavour interactions, denoted g_{ic} and g_{id}, where the flavour c is treated as p-type and d as m-type. The interflavour vertices do not require this distinction. The most general set of interactions between patches/flavours allowed by momentum conservation then results in a 20 coupling model,

$$
V = \frac{1}{2}\sum_{\alpha,\beta}\left[V_{g,c}+V_{g,d}+V_h+V_j+V_l\right], \tag{S2}
$$

$$
\begin{aligned}
V_{g,c} &= g_{1c}c^\dagger_\alpha c^\dagger_\beta c_\alpha c_\beta + g_{2c}c^\dagger_\alpha c^\dagger_\beta c_\beta c_\alpha + g_{3c}c^\dagger_\alpha c^\dagger_\alpha c_\beta c_\beta + \frac{1}{2}g_{4c}c^\dagger_\alpha c^\dagger_\alpha c_\alpha c_\alpha, \\
V_{g,d} &= g_{1d}d^\dagger_\alpha d^\dagger_\beta d_\alpha d_\beta + g_{2d}d^\dagger_\alpha d^\dagger_\beta d_\beta d_\alpha + g_{3d}d^\dagger_\alpha d^\dagger_\alpha d_\beta d_\beta + \frac{1}{2}g_{4d}d^\dagger_\alpha d^\dagger_\alpha d_\alpha d_\alpha, \\
V_h &= h_1 c^\dagger_\alpha d^\dagger_\beta d_\alpha c_\beta + h_2 c^\dagger_\alpha d^\dagger_\beta d_\beta c_\alpha + h_3 c^\dagger_\alpha d^\dagger_\alpha d_\beta c_\beta + \frac{1}{2}h_4 c^\dagger_\alpha d^\dagger_\alpha d_\alpha c_\alpha + (c \leftrightarrow d), \\
V_j &= j_1 d^\dagger_\alpha d^\dagger_\beta c_\alpha c_\beta + j_2 d^\dagger_\alpha d^\dagger_\beta c_\beta c_\alpha + j_3 d^\dagger_\alpha d^\dagger_\alpha c_\beta c_\beta + \frac{1}{2}j_4 d^\dagger_\alpha d^\dagger_\alpha c_\alpha c_\alpha + (c \leftrightarrow d), \\
V_l &= l_1 d^\dagger_\alpha c^\dagger_\beta d_\alpha c_\beta + l_2 d^\dagger_\alpha c^\dagger_\beta d_\beta c_\alpha + l_3 d^\dagger_\alpha c^\dagger_\alpha d_\beta c_\beta + \frac{1}{2}l_4 d^\dagger_\alpha c^\dagger_\alpha d_\alpha c_\alpha + (c \leftrightarrow d).
\end{aligned}
$$

For honeycomb systems $g_{ic} = g_{id}$, reducing the number of independent couplings to 16. The corresponding Feynman diagrams are presented in Figure 1(d) [of the main text]. Due to the large density of states near the TvHS, the Coulomb repulsion is expected to be strongly screened, and we therefore model these interactions as momentum independent.

B. Tight binding model

To estimate the bare interactions, we start from the output of DFT results and directly compute the interaction vertices. The interacting tight binding model of [1] gives

$$
H_{int} = U\sum_{i,\sigma,\mu} n_{i\sigma\mu\uparrow}n_{i\sigma\mu\downarrow} + U'\sum_{i,\sigma,\mu<\mu',s,s'} n_{i\sigma\mu s}n_{i\sigma\mu' s'} + V\sum_{\langle ij\rangle\sigma\sigma'\mu\mu'} n_{i\sigma\mu s}n_{j\sigma'\mu' s'} + J\sum_{i\sigma\mu<\mu',s,s'} c^\dagger_{i\sigma\mu s}c^\dagger_{i\sigma\mu' s'}c_{i\sigma\mu s'}c_{i\sigma\mu' s} + J'\sum_{i\sigma\mu\neq\mu'} c^\dagger_{i\sigma\mu\uparrow}c^\dagger_{i\sigma\mu\downarrow}c_{i\sigma\mu'\downarrow}c_{i\sigma\mu'\uparrow} \tag{S3}
$$

where i enumerates unit cells, $\sigma = A, B, C$ enumerates sublattice, $\mu = \pm$ enumerates the orbital index, and s is spin. Ref. [1] finds $U \sim 1-2$ eV, $U' = 0.8U$, $V \approx 0.3U$ and $J = J' = 0.1U$.

TABLE S1: Estimates of the bare coupling values in the patch model S2 for AV_3Sb_5. Projecting the pure and mixed sublattice form factors onto the cRPA results of Ref. [1] results in the below values, where the intra-orbital, inter-orbital, Hund's, pair hopping, and nearest neighbour repulsions are $U =$ 1-2 eV, with $U' = 0.8U$, $J = J' = 0.1U$ and $V = 0.3U$.

	$g_{i,c}$	$g_{i,d}$	h_i	j_i	l_i
$i = 1$	0	$\frac{1}{4}(U + V)$	0	0	$\frac{1}{2}J$
$i = 2$	V	$\frac{1}{4}U + V$	$\frac{1}{2}U' + V$	0	0
$i = 3$	0	$\frac{1}{4}(U + V)$	0	$\frac{1}{2}J'$	0
$i = 4$	$U + V$	$\frac{1}{2}U + V$	V	0	0

TABLE S2: Initial conditions leading to the phase diagram of Figure 4(a)ii [of the main text]. Taking $U = 1.3$ eV, with ratios fixed at $U' = 0.8U$, $J = J' = 0.1U$ and $V = 0.3U$ as per Ref. [1] and Table S1. Blue entries differ from the initial conditions of Table S1.

	$g_{i,c}$	$g_{i,d}$	h_i	j_i	l_i
$i = 1$	$-\frac{2}{3}V$	$\frac{1}{4}(U + V)$	$-\frac{7}{4}V$	0	$\frac{1}{2}J$
$i = 2$	V	$\frac{1}{4}U + V$	$\frac{1}{2}U' + V$	0	0
$i = 3$	0	$\frac{1}{4}(U + V)$	0	$\frac{1}{2}J'$	0
$i = 4$	$U + V$	$\frac{1}{2}U + V$	V	0	0

C. Patch Model – initial coupling estimates

Here we list the estimates for the bare couplings (which serve as initial conditions for RG flow). We evaluate with the spin structure $s, s; -s, -s$. Before evaluating the orbital form factors, we note the orbital selection rules (i.e. conditions on index i in (S3)) imply

$$g_{i\nu} \propto U, V, \qquad h_i \propto U', V, \qquad j_i \propto J', \qquad l_i \propto J. \tag{S4}$$

Including form factors, evaluated at the M-points, we arrive at the initial conditions of Table I [of the main text], repeated here as Table S1. The initial conditions taken to arrive at the phase diagram of Figure 4(a)ii and 4(b)ii [of the main text] are presented in Table S2 and S3, respectively.

TABLE S3: Initial conditions leading to the phase diagram of Figure 4(b)ii [of the main text]. Again taking $U = 1.3$ eV, with ratios fixed at $U' = 0.8U$, $J = J' = 0.1U$ and $V = 0.3U$. Blue entries differ from the initial conditions of Table S1

.

	$g_{i,c}$	$g_{i,d}$	h_i	j_i	l_i
$i = 1$	$-\frac{2}{3}V$	$\frac{1}{4}(U+V)$	$-\frac{7}{4}V$	0	$\frac{1}{2}J$
$i = 2$	V	$\frac{1}{4}U+V$	$\frac{1}{2}U'+V$	0	0
$i = 3$	0	0	0	$\frac{1}{2}J'$	0
$i = 4$	$U+V$	$\frac{1}{2}U+V$	V	0	0

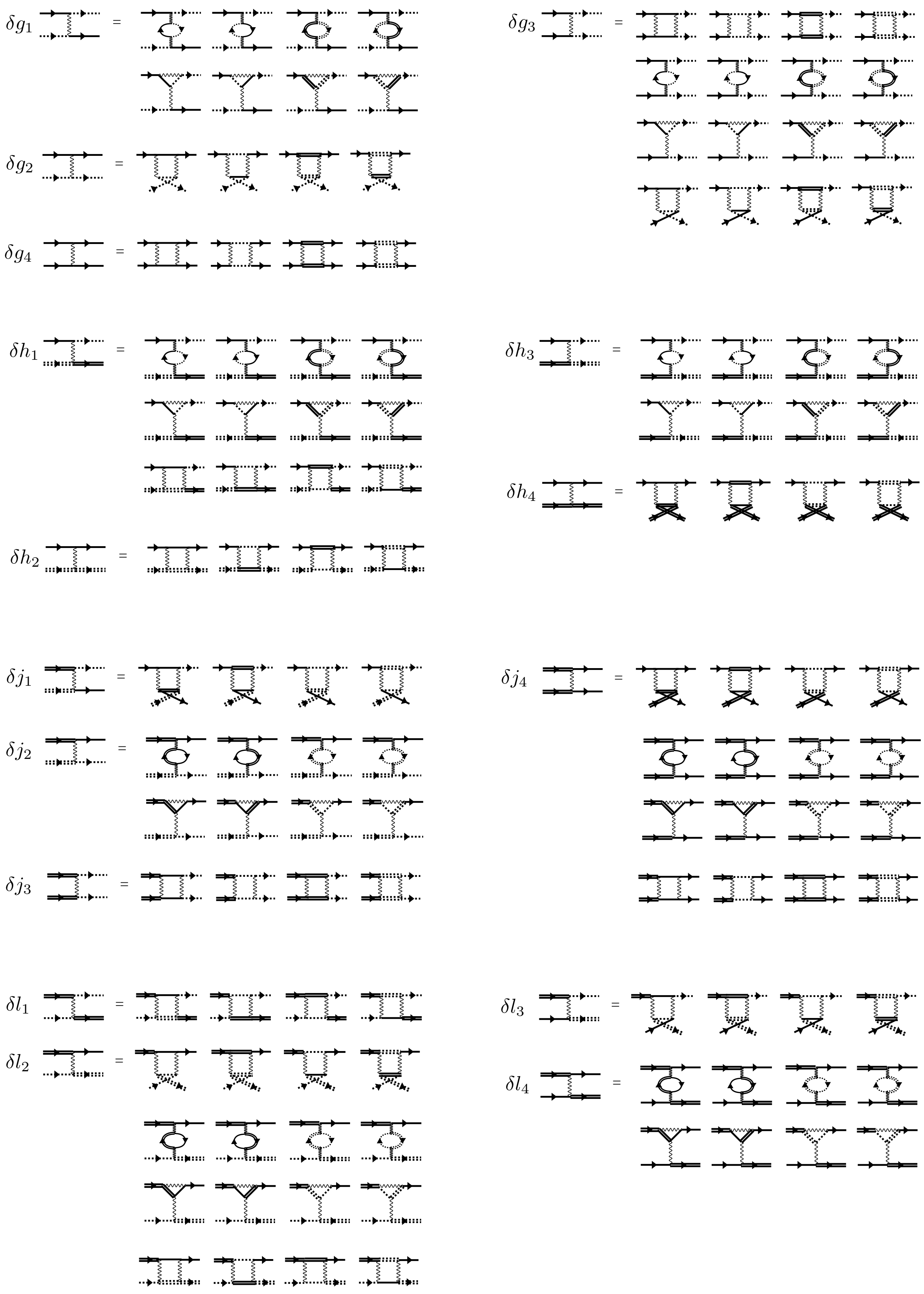

FIG. S1: **Flow equations for the couplings**: The distinct Feynman diagrams representing leading logarithmic ($\log^2$) corrections to the couplings (S2). Flavour indices on g_{ic} and g_{id} are suppressed. The explicit flow equations for the couplings $g_{i\nu}, h_i, j_i, l_i$ are given by (S7).

II. FLOW EQUATIONS FOR THE COUPLINGS

To $\log^2$ accuracy, the topological distinct Feynman diagrams that contribute to the β functions are presented in Figure S1. Owing to the momentum independence of the contact interactions, the bubble, vertex, and box corrections depicted in Figure S1 all reduce to simple two-particle susceptibilities. We define the particle-particle and particle-hole susceptibilities,

$$\Pi_{pp}^{\nu\nu'}(\boldsymbol{q}) = -i\int \mathrm{Tr}\; G_\nu(\omega, \boldsymbol{p}+\boldsymbol{q})G_{\nu'}(-\omega,-\boldsymbol{p})\; \tfrac{d\omega d^2\boldsymbol{p}}{(2\pi)^3}, \qquad \Pi_{ph}^{\nu\nu'}(\boldsymbol{q}) = -i\int \mathrm{Tr}\; G_\nu(\omega, \boldsymbol{p}+\boldsymbol{q})G_{\nu'}(\omega,\boldsymbol{p})\; \tfrac{d\omega d^2\boldsymbol{p}}{(2\pi)^3} \tag{S5}$$

where $G_\nu(\omega,\boldsymbol{p})$ is the fermion Green's function for the flavour ν, and pp or ph denote particle-particle or particle-hole. The RG time is given by $t = \Pi_{pp}^{cc}(\boldsymbol{0})$, and the d_i nesting factors are defined as

$$d_0 = \frac{d}{dt}\Pi_{pp}^{dd}(\boldsymbol{0}), \qquad d_1 = \frac{d}{dt}\Pi_{pp}^{cd}(\boldsymbol{Q}_\alpha), \qquad d_2 = \frac{d}{dt}\Pi_{ph}^{cc}(\boldsymbol{Q}_\alpha), \qquad d_3 = \frac{d}{dt}\Pi_{ph}^{dd}(\boldsymbol{Q}_\alpha), \qquad d_4 = \frac{d}{dt}\Pi_{ph}^{cd}(\boldsymbol{0}). \tag{S6}$$

They are plotted in Figure S2 against the infrared scale given by the temperature T.

The explicit β functions are,

$$\begin{aligned}
\dot{g}_{1c} &= 2d_2 g_{1c}\left(g_{2c}-g_{1c}\right) - 2d_3\left(-h_1 l_2 + h_3\left(h_3 - l_3\right) + h_1^2\right),\\
\dot{g}_{2c} &= d_2\left(g_{2c}^2 + g_{3c}^2\right) + d_3\left(l_2^2 + l_3^2\right),\\
\dot{g}_{3c} &= -2d_2\left(g_{1c} - 2g_{2c}\right)g_{3c} + 2d_3 h_3 l_2 + 2d_3 h_1 l_3 - 4d_3 h_1 h_3 - d_0 j_3^2 - 2d_0 j_3 j_4 + 2d_3 l_2 l_3 - g_{3c}^2 - 2g_{4c}g_{3c},\\
\dot{g}_{4c} &= -d_0\left(2j_3^2 + j_4^2\right) - 2g_{3c}^2 - g_{4c}^2,\\
\dot{g}_{1d} &= 2d_3 g_{1d}\left(g_{2d}-g_{1d}\right) - 2d_2\left(-h_1 l_2 + h_3\left(h_3 - l_3\right) + h_1^2\right),\\
\dot{g}_{2d} &= d_3\left(g_{2d}^2 + g_{3d}^2\right) + d_2\left(l_2^2 + l_3^2\right),\\
\dot{g}_{3d} &= -d_0 g_{3d}\left(g_{3d} + 2g_{4d}\right) - 2d_3\left(g_{1d} - 2g_{2d}\right)g_{3d} + 2d_2 h_3 l_2 + 2d_2 h_1 l_3 - 4d_2 h_1 h_3 + 2d_2 l_2 l_3 - j_3^2 - 2j_4 j_3,\\
\dot{g}_{4d} &= -d_0\left(2g_{3d}^2 + g_{4d}^2\right) - 2j_3^2 - j_4^2,\\
\dot{h}_1 &= d_2\left(g_{1c}\left(l_2 - 2h_1\right) + g_{3c}\left(l_3 - h_3\right) + h_1 g_{2c}\right) + d_3\left(g_{1d}\left(l_2 - 2h_1\right) + g_{3d}\left(l_3 - h_3\right) + h_1 g_{2d}\right) + d_4 h_1^2 - 2d_1 h_2 h_1 + 2d_4 h_4 h_1 + d_4 j_1^2 + 2d_4 j_1 j_4 - 2d_1 l_1 l_2,\\
\dot{h}_1 &= d_2\left(g_{1c}\left(l_2 - 2h_1\right) + g_{3c}\left(l_3 - h_3\right) + h_1 g_{2c}\right) + d_3\left(g_{1d}\left(l_2 - 2h_1\right) + g_{3d}\left(l_3 - h_3\right) + h_1 g_{2d}\right) + d_4(h_1^2 + 2h_4 h_1 + j_1^2 + 2j_1 j_4) - 2d_1(h_2 h_1 + l_1 l_2),\\
\dot{h}_2 &= -d_1\left(h_1^2 + h_2^2 + l_1^2 + l_2^2\right),\\
\dot{h}_3 &= d_2\left(g_{3c}\left(l_2 - h_1\right) + g_{1c}\left(l_3 - 2h_3\right) + h_3 g_{2c}\right) + d_3\left(g_{3d}\left(l_2 - h_1\right) + g_{1d}\left(l_3 - 2h_3\right) + h_3 g_{2d}\right),\\
\dot{h}_4 &= d_4\left(2h_1^2 + h_4^2 + 2j_1^2 + j_4^2\right),\\
\dot{j}_1 &= 2d_4\left(h_4 j_1 + h_1\left(j_1 + j_4\right)\right),\\
\dot{j}_2 &= 2d_4\left(h_4 j_2 + h_1\left(j_2 + j_4\right) - 2j_2 l_2 - 2j_2 l_4 + j_1 l_2 - j_4 l_2 + j_1 l_4\right),\\
\dot{j}_3 &= -d_0\left(\left(j_3 + j_4\right)g_{3d} + j_3 g_{4d}\right) + j_3\left(-g_{4c}\right) - \left(j_3 + j_4\right)g_{3c},\\
\dot{j}_4 &= -d_0\left(2j_3 g_{3d} + j_4 g_{4d}\right) + d_4\left(4h_1\left(j_1 + j_2\right) + 4h_4 j_4 + 4j_1 l_2 - 8j_2 l_2 - 2j_4 l_4\right) - 2j_3 g_{3c} - j_4 g_{4c},\\
\dot{l}_1 &= -2d_1\left(h_2 l_1 + h_1 l_2\right),\\
\dot{l}_2 &= d_2 l_2 g_{2c} + d_2 l_3 g_{3c} + d_3 l_2 g_{2d} + d_3 l_3 g_{3d} + 2d_4\left(h_1 l_2 + h_4 l_2 + h_1 l_4 - j_2^2 - j_4 j_2 + j_1\left(j_2 + j_4\right) - l_2^2 - 2l_2 l_4\right) - 2d_1\left(h_1 l_1 + h_2 l_2\right),\\
\dot{l}_3 &= d_2\left(l_2 g_{3c} + l_3 g_{2c}\right) + d_3\left(l_2 g_{3d} + l_3 g_{2d}\right),\\
\dot{l}_4 &= 2d_4\left(2h_1 l_2 + h_4 l_4 - 2j_2^2 + 2j_1 j_2 - 2l_2^2 - l_4^2\right).
\end{aligned} \tag{S7}$$

Identifying $g_{ic} = g_{id}$, as appropriate for a TvHS in a honeycomb system, the β functions reduce to 16 independent functions, which may be obtained straightforwardly from the above expressions.

The RG flow of the couplings, i.e. solutions to (S7), are presented in Figure S2. For illustration, the initial conditions are taken from Table S2.

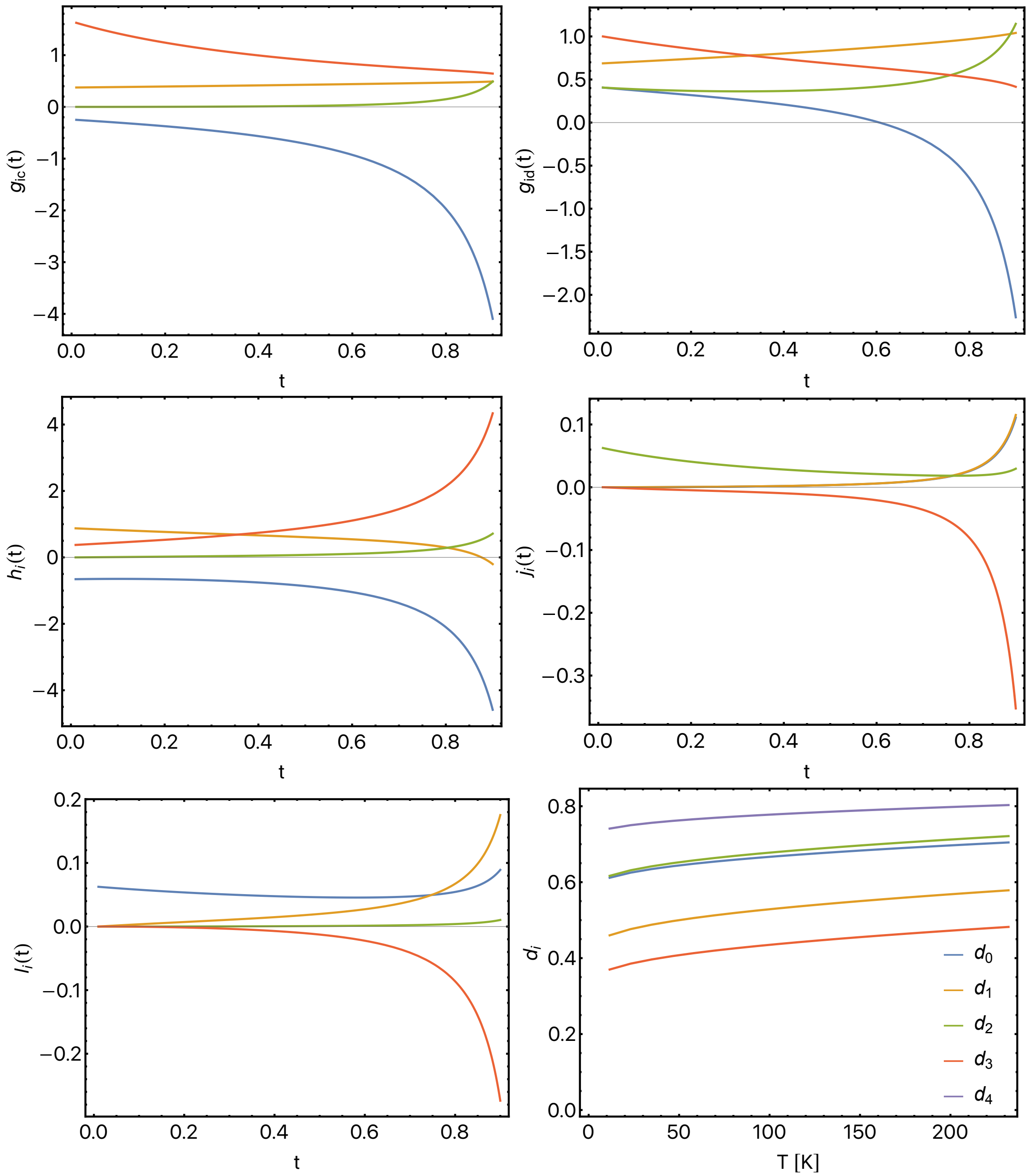

FIG. S2: RG flow of the couplings obtained by integration of the flow equations (S7), with initial conditions taken from Table S2. Here {blue, orange, green, red} corresponds to $i = \{1, 2, 3, 4\}$. Also included are the d_i-factors which appear in the RG analysis (S7) and (S8), plotted as a function of T.

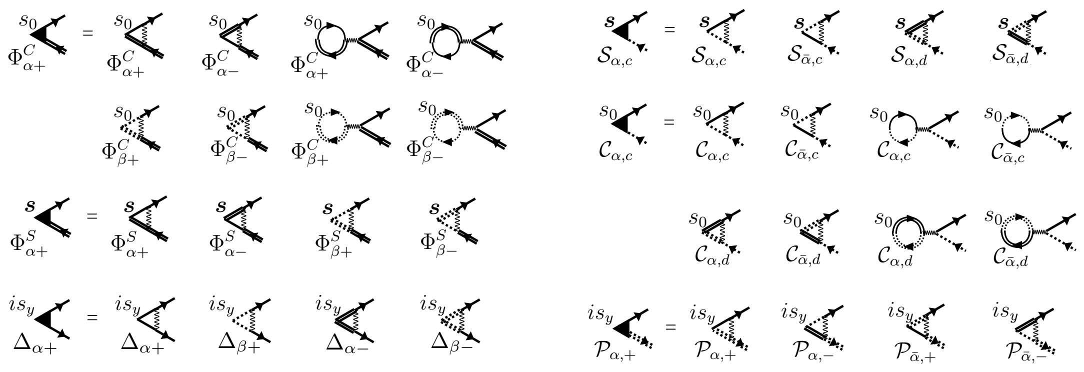

FIG. S3: **Gap equations**: The Feynman diagrams representing the leading logarithmic corrections to the order parameter fields are shown. The result is a set of linear equations for the susceptibilities, which give the *gap equations* for the various possible order parameters, in Eq. (S8).

III. ORDER PARAMETER GAP EQUATIONS

The flow equations for the order parameter vertices $\mathcal{O}_i = \{\mathcal{S}_{\alpha i}, \mathcal{C}_{\alpha i}, \Delta_{\alpha i}, \mathcal{P}_{\alpha\pm}, \Phi^C_{\alpha\pm}, \Phi^S_{\alpha\pm}\}$ referenced in the main text are represented diagrammatically in Fig. S3 and are given explicitly by (repeated here for convenience)

$$\begin{aligned}
\frac{\partial}{\partial t}\Phi^C_{\alpha+} &= d_4 \sum_{\beta\neq\alpha}\Big\{(h_4-2l_4)\Phi^C_{\alpha+} - j_4\Phi^C_{\alpha-} + (h_1-2l_2)\Phi^C_{\beta+} + (j_1-2j_2)\Phi^C_{\beta-}\Big\}\\
\frac{\partial}{\partial t}\Phi^S_{\alpha+} &= d_4 \sum_{\beta\neq\alpha}\Big\{h_4\Phi^S_{\alpha+} + j_4\Phi^S_{\alpha-} + h_1\Phi^S_{\beta+} + j_1\Phi^S_{\beta-}\Big\}\\
\frac{\partial}{\partial t}\mathcal{P}_{\alpha,+} &= -d_1\Big\{h_2\mathcal{P}_{\alpha,+} + h_1\mathcal{P}_{\alpha,-} + l_1\mathcal{P}_{\bar\alpha,+} + l_2\mathcal{P}_{\bar\alpha,-}\Big\}\\
\frac{\partial}{\partial t}\mathcal{C}_{\alpha,\nu} &= d_{2\nu}(g_{2,\nu}-2g_{1,\nu})\mathcal{C}_{\alpha,\nu} - d_{2\nu}g_{3,\nu}\mathcal{C}_{\bar\alpha,\nu} + d_{2\bar\nu}(l_2-2h_1)\mathcal{C}_{\alpha,\bar\nu} + d_{2\bar\nu}(l_3-2h_3)\mathcal{C}_{\bar\alpha,\bar\nu}\\
\frac{\partial}{\partial t}\mathcal{S}_{\alpha,\nu} &= d_{2\nu}g_{2,c}\mathcal{S}_{\alpha,\nu} + d_{2\nu}g_{3,c}\mathcal{S}_{\bar\alpha,\nu} + d_{2\bar\nu}l_2\mathcal{S}_{\alpha,\bar\nu} + d_{2\bar\nu}l_3\mathcal{S}_{\bar\alpha,\bar\nu}\\
\frac{\partial}{\partial t}\Delta_{\alpha,\nu} &= -\sum_{\beta\neq\alpha}\Big\{d_{0\bar\nu}g_{4,c}\Delta_{\alpha,c} + d_{0\bar\nu}g_{3,c}\Delta_{\beta,c} + d_{0\nu}j_4\Delta_{\alpha,d} + d_{0\nu}j_3\Delta_{\beta,d}\Big\}
\end{aligned} \tag{S8}$$

with indices as defined previously—c, d, $\pm$ referring to flavour, α to patch—and $\bar\alpha$ denoting the patch connected to α by a nesting vector. To make the equations compact, we have introduced $\nu = \{c, d\}$ with $\bar\nu = \{d, c\}$, and combined the d-factors such that $d_{0c} = 1, d_{0d} = d_0, d_{2c} = d_2, d_{2d} = d_3$, and we approximate $\partial_t d_i = 0$, as stated in the Methods section. The couplings entering the gap equations are understood to inherit scale-dependence from the coupling RG equations (S7). The eigenvectors for this linear system of gap equations give the possible order parameter structures, and those with the largest eigenvalue are the leading instabilities. The eigenvalues of (S8) are lengthy, unenlightening expressions so we have chosen not to present them.

The RG flow of the solutions to (S8) are present in Figure S4, subject to the initial conditions of Table S2 and S3.

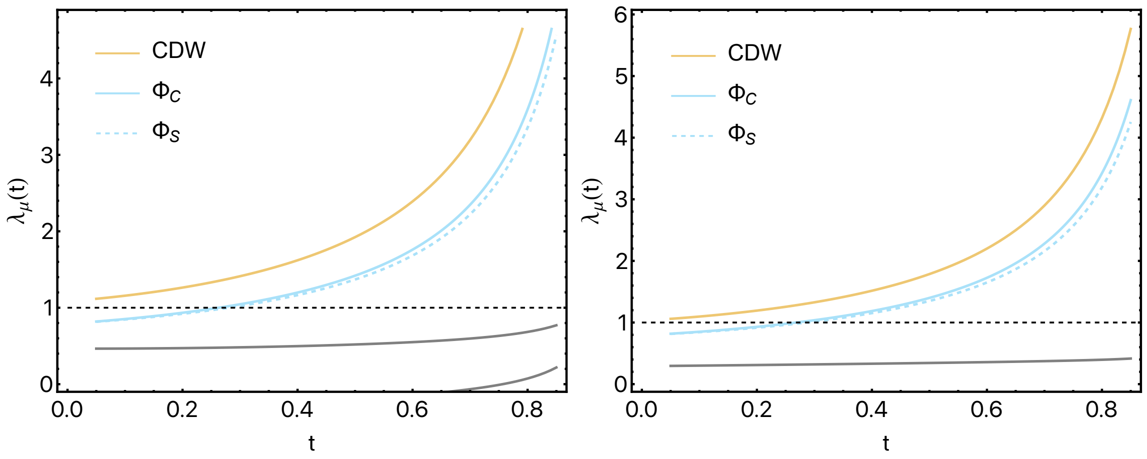

FIG. S4: Order parameter eigenvalue flow evaluated from the gap equations (S8), with initial conditions for (a) taken from Table S2, and for (b) from S3. The CDW and $\Phi_{C,S}$ eigenvalues are marked, all other eigenvalues are subleading, and plotted in grey.

IV. RG FIXED RAYS

Integrating the flow equations, one finds that the couplings generically diverge, resulting in an instability. For a given set of initial conditions, the couplings approach fixed ratios of each other at long RG times – referred to as an RG 'fixed ray', 'fixed trajectory', or sometimes more loosely refered to as a 'fixed point'.

To obtain the RG fixed rays, we insert the scaling form $\{g_{i\nu}, h_i, j_i, l_i\} = \{G_{i\nu}, H_i, J_i, L_i\}\mathtt{S}$, with $\mathtt{S} = 1/(t_c - t)$, into the RG equations which at long RG times $t \to t_c$ allows the differential flow equations (S7) to be reduced to nonlinear algebraic equations for the t-independent coefficients $\{G_{i\nu}, H_i, J_i, L_i\}$. We summarise the procedure, which has been discussed in detail elsewhere, c.f Ref. [2]:

1. Let us denote the set of running couplings as $\gamma_i = \{g_{i\nu}, h_i, j_i, l_i\}$ and the set of scaling coefficients $\Gamma_i = \{G_{i\nu}, H_i, J_i, L_i\}$.

2. The fixed rays are found via $\dot{\Gamma}_i = \left[\dot{\gamma}_i - \Gamma_i\dot{\mathtt{S}}\right]/\mathtt{S} = \left[\beta_i[\{\Gamma\}] - \Gamma_i\right]\mathtt{S} = 0$.

3. To analyse the stability of the fixed points, we examine the matrix

$$T_{ij} = \frac{\partial}{\partial\gamma_j}\left(\beta_i[\{\Gamma\}] - \Gamma_i\right). \tag{S9}$$

   Evaluating T_{ij} at the fixed points (i.e. at the solutions to $\Gamma_i = \beta_i[\Gamma_j]$), we discard those fixed points with greater than one positive eigenvalues. The stable fixed rays satisfy this condition.

We have employed a slightly different approach to Ref. [2], which we found to be more efficient for the present problem. Ref. [2] eliminate one coupling g_i by using it as a proxy for the RG time. In our case we do not eliminate any g_i and retain all fixed points with just one unstable direction, which may similarly be taken as a proxy for the RG time; our approach reproduces the results of [2], i.e. in the limit of no interflavour couplings.

We note that the fixed rays featuring CDW order found in that work are in fact unstable to the addition of interflavour coupling, as evidenced by the fact our model has no fixed rays with charge order. Charge ordering is therefore not a dominant weak coupling instability, as discussed in the main text, and must set in at shorter RG times before fixed ray behaviour sets in, requiring the bare couplings to be adequately large. Below we compute the stable fixed rays; we make use of the result that true weak-coupling instabilities require the associated order parameter eigenvalue coefficient $\Lambda_i \geq 1/2$ [3], where the eigenvalue is $\lambda_i = \Lambda_i\mathtt{S}$.

A. Model: Kagome $\kappa = 2$

In this model, there are eight unique stable fixed rays. Including only the non-zero terms for brevity, the fixed ray scaling coefficients (i.e. $\{g_{i\nu}, h_i, j_i, l_i\} = \{G_{i\nu}, H_i, J_i, L_i\}$S) are

$$
\begin{aligned}
&\mathbf{1}.\ \big\{G_{2c} = 0.00247, G_{3c} = 0.06035, G_{4c} = -0.18739, G_{2d} = 0.00356, G_{3d} = 0.09047, G_{4d} = -0.28107,\\
&\quad H_1 = -0.00326, H_3 = 0.00026, H_4 = 0.18837, J_1 = 0.00324, J_2 = 0.00324, J_3 = 0.07354, J_4 = -0.45461\big\}\\
&\mathbf{2}.\ \big\{G_{2c} = 0.00694, G_{4c} = -0.01131, G_{2d} = 0.01082, G_{4d} = -0.01697,\\
&\quad H_2 = -0.00843, H_4 = 0.01318, J_2 = 0.12316, J_4 = 0.12953, L_2 = -0.12606, L_4 = -0.12248\big\}\\
&\mathbf{3}.\ \big\{G_{2c} = 0.00694, G_{4c} = -0.01131, G_{2d} = 0.01082, G_{4d} = -0.01697,\\
&\quad H_2 = -0.00843, H_4 = 0.01318, J_2 = -0.12316, J_4 = -0.12953, L_2 = -0.12606, L_4 = -0.12248\big\}\\
&\mathbf{4}.\ \big\{G_{1c} = -0.01244, G_{2c} = 0.00303, G_{4c} = -0.05957, G_{1d} = -0.01939, G_{2d} = 0.00472, G_{4d} = -0.08937, H_1 = 0.16721,\\
&\quad H_2 = -0.01876, H_4 = 0.17096, J_1 = -0.15909, J_2 = -0.07964, J_4 = -0.2899, L_1 = -0.01504, L_2 = 0.08341, L_4 = 0.05094\big\}\\
&\mathbf{5}.\ \big\{G_{2d} = 0.12733, G_{3d} = 0.52591, G_{4d} = -0.84684\big\}\\
&\mathbf{6}.\ \big\{G_{2c} = 0.08906, G_{3c} = 0.3514, G_{4c} = -0.55499\big\}\\
&\mathbf{7}.\ \big\{G_{2c} = 0.06738, G_{3c} = -0.30805, G_{4c} = -0.25463\big\}\\
&\mathbf{8}.\ \big\{G_{2d} = 0.09823, G_{3d} = -0.465, G_{4d} = -0.38935\big\}.
\end{aligned}
\tag{S10}
$$

At these fixed rays the following ordered phases are supported, respectively:

$$
\mathbf{1}.\ \Delta_d + \Phi_d^C + \Phi_d^S,\quad \mathbf{2}.\ \Phi_s^C,\quad \mathbf{3}.\ \Phi_s^C,\quad \mathbf{4}.\ \Phi_s^S,\quad \mathbf{5}.\ \Delta_d,\quad \mathbf{6}.\ \Delta_d,\quad \mathbf{7}.\ \Delta_d,\quad \mathbf{8}.\ \Delta_d. \tag{S11}
$$

B. Model: Kagome $\kappa = 1$

In this model, there are seven unique stable fixed rays. The corresponding nonzero couplings are

$$
\begin{aligned}
&\mathbf{1}.\ \big\{G_{1c} = -0.00077, G_{2c} = 0.00038, G_{3c} = 0.02784, G_{4c} = -0.16242, G_{1d} = -0.00077, G_{2d} = 0.00038, G_{3d} = 0.02784,\\
&\quad G_{4d} = -0.16242, H_1 = -0.02784, H_2 = -0.00038, H_3 = 0.00077, H_4 = 0.16242, J_1 = 0.02778, J_2 = 0.02778,\\
&\quad J_3 = 0.02778, J_4 = -0.36461\big\}\\
&\mathbf{2}.\ \big\{G_{2c} = 0.00478, G_{4c} = -0.01034, G_{2d} = 0.00478, G_{4d} = -0.01034,\\
&\quad H_2 = -0.00478, H_4 = 0.01034, J_2 = -0.09656, J_4 = -0.10118, L_2 = -0.09773, L_4 = -0.09587\big\}\\
&\mathbf{3}.\ \big\{G_{1c} = -0.00841, G_{2c} = 0.00208, G_{4c} = -0.05558, G_{1d} = -0.00841, G_{2d} = 0.00208, G_{4d} = -0.05558, H_1 = 0.12905,\\
&\quad H_2 = -0.0105, H_4 = 0.13597, J_1 = -0.12584, J_2 = -0.06292, J_4 = -0.22912, L_1 = -0.00841,\\
&\quad L_2 = 0.06452, L_4 = 0.04019\big\}\\
&\mathbf{4}.\ \{G_{2d} = 0.05258, G_{3d} = -0.32, G_{4d} = -0.28741\}\\
&\mathbf{5}.\ \{G_{2d} = 0.06129, G_{3d} = 0.34472, G_{4d} = -0.61106\}\\
&\mathbf{6}.\ \{G_{2c} = 0.05258, G_{3c} = -0.32, G_{4c} = -0.28741\}\\
&\mathbf{7}.\ \{G_{2c} = 0.06129, G_{3c} = 0.34472, G_{4c} = -0.61106\}\,.
\end{aligned}
\tag{S12}
$$

These fixed rays support the following ordered phases:

$$
\mathbf{1}.\ \Phi_d^C + \Delta_d + \Phi_d^S,\quad \mathbf{2}.\ \Phi_s^C,\quad \mathbf{3}.\ \Phi_s^S,\quad \mathbf{4}.\ \Delta_d,\quad \mathbf{5}.\ \Delta_d,\quad \mathbf{6}.\ \Delta_d,\quad \mathbf{7}.\ \Delta_d. \tag{S13}
$$

C. Model: Honeycomb $\kappa = 1$

In this model, there are three unique stable fixed rays. The corresponding nonzero couplings are

$$\begin{aligned}
&\mathbf{1}.\ \big\{G_2 = 0.00316, G_4 = -0.13371, H_2 = -0.00316, H_4 = 0.13371, J_2 = 0.07916, J_4 = -0.34034,\\
&\quad L_2 = 0.07952, L_4 = -0.09172\big\}\\
&\mathbf{2}.\ \big\{G_2 = 0.00479, G_4 = -0.01017, H_2 = -0.00479, H_4 = 0.01017, J_2 = -0.09694, J_4 = -0.10033,\\
&\quad L_2 = -0.09783, L_4 = -0.09645\big\}\\
&\mathbf{3}.\ \{G_2 = 0.05258, G_3 = -0.32, G_4 = -0.28741\}\,. \qquad \text{(S14)}
\end{aligned}$$

At these fixed rays the following ordered phases are supported:

$$\mathbf{1}.\ \Phi_d^C,\ \mathbf{2}.\ \Phi_s^C,\ \ \mathbf{3}.\ \Delta_d. \qquad \text{(S15)}$$

V. FREE ENERGY EXPANSION

Here we provide the explicit details of the free energy expansion. We wish to calculate the free energy

$$\mathcal{F} = \tfrac{1}{2\lambda_\Phi}\sum_i |\Phi_i|^2 + \tfrac{1}{2}\sum_{\alpha\neq\beta;\nu=c,d} \mathcal{V}^{-1}_{\nu\nu'}\, C_{\alpha\beta\nu}C^*_{\alpha\beta\nu'} + \tfrac{1}{2}\mathrm{Tr}(\mathcal{G}_0 M)^2 - \tfrac{1}{3}\mathrm{Tr}(\mathcal{G}_0 M)^3 + \tfrac{1}{4}\mathrm{Tr}(\mathcal{G}_0 M)^4, \tag{S16}$$

with order parameter matrix M and Green's function $\mathcal{G}_0$ as defined in the Methods. Writing the contributions as $\mathcal{F} = \mathcal{F}_0 + \mathcal{F}_2 + \mathcal{F}_3 + \mathcal{F}_4$;

$$\mathcal{F}_2 = \tfrac{1}{2\lambda_\Phi}\sum_i |\Phi_i|^2 + \tfrac{1}{2}\sum_{\alpha\neq\beta;\nu=c,d} \mathcal{V}^{-1}_{\nu\nu'}\, C_{\alpha\beta\nu}C^*_{\alpha\beta\nu'} + \tfrac{1}{2}\mathrm{Tr}(\mathcal{G}_0 M)^2 \tag{S17}$$

$$\mathcal{F}_3 = -\tfrac{1}{3}\mathrm{Tr}(\mathcal{G}_0 M)^3 \tag{S18}$$

$$\mathcal{F}_4 = \tfrac{1}{4}\mathrm{Tr}(\mathcal{G}_0 M)^4 \tag{S19}$$

Quadratic term. Expanding the trace in Eq. (S17),

$$\tfrac{1}{2}\mathrm{Tr}(\mathcal{G}_0 M)^2 = a_c \sum_{\alpha\neq\beta} |C_{\alpha\beta c}|^2 + a_d \sum_{\alpha\neq\beta} |C_{\alpha\beta d}|^2 + a_\Phi \left(|\Phi_a|^2 + |\Phi_b|^2\right) \tag{S20}$$

where

$$a_c = \sum_n \int \mathcal{G}_{ci}(i\omega_n, \boldsymbol{q})\mathcal{G}_{cj}(i\omega_n, \boldsymbol{q}), \qquad a_d = \sum_n \int \mathcal{G}_{di}(i\omega_n, \boldsymbol{q})\mathcal{G}_{dj}(i\omega_n, \boldsymbol{q}), \qquad a_\Phi = \sum_n \int \mathcal{G}_{ci}(i\omega_n, \boldsymbol{q})\mathcal{G}_{di}(i\omega_n, \boldsymbol{q}). \tag{S21}$$

The coefficients a_i are evaluated in Figure S5(a).

Cubic term. The cubic term we find from Eq. (S18) is

$$-\mathcal{F}_3 = \tfrac{1}{3}\mathrm{Tr}(\mathcal{G}_0 M)^3 = b_c(C_{12c}C_{23c}C_{31c} + \text{c.c.}) + b_d(C_{12d}C_{23d}C_{31d} + \text{c.c.}) \tag{S22}$$

where

$$b_c = \sum_n \int \mathcal{G}_{c1}(i\omega_n, \boldsymbol{q})\mathcal{G}_{c2}(i\omega_n, \boldsymbol{q})\mathcal{G}_{c3}(i\omega_n, \boldsymbol{q}), \qquad b_d = \sum_n \int \mathcal{G}_{d1}(i\omega_n, \boldsymbol{q})\mathcal{G}_{d2}(i\omega_n, \boldsymbol{q})\mathcal{G}_{d3}(i\omega_n, \boldsymbol{q}). \tag{S23}$$

The coefficients b_ν are evaluated in Figure S5(b) for $\kappa = 2$. We consider two cases: rCDW and iCDW, corresponding to $\Phi = 0, \pi/2$, respectively. For iCDW the cubic terms vanish, while for rCDW it is non-zero. Hence, in general, the cubic term favours rCDW.

Quartic term. Lastly, we arrive at the quartic term from expanding the trace in Eq. (S19). We first write the result in terms of Φ_α where α indexes patch, and then convert to the d-wave basis $\Phi_{a,b}$:

$$\begin{aligned}\mathcal{F}_4 = \tfrac{1}{4}\mathrm{Tr}(\mathcal{G}_0 M)^4 = {}& \tfrac{1}{2}c_{1c}\sum_{\alpha\neq\beta}|C_{\alpha\beta c}|^4 + \tfrac{1}{2}c_{1d}\sum_{\alpha\neq\beta}|C_{\alpha\beta d}|^4 + c_{2c}\sum_{\alpha\neq\beta\neq\gamma}|C_{\alpha\beta c}|^2|C_{\alpha\gamma c}|^2 + c_{4d}\sum_{\alpha\neq\beta\neq\gamma}|C_{\alpha\beta d}|^2|C_{\alpha\gamma d}|^2 + \tfrac{1}{2}c_\Phi\sum_\alpha|\Phi_\alpha|^4 \\ &+ c_3\sum_{\alpha\neq\beta}(C_{\alpha\beta d}C^*_{\alpha\beta c}\Phi_\alpha\Phi^*_\beta + \text{c.c.}) + c_{4c}\sum_{\alpha\neq\beta}|C_{\alpha\beta c}|^2(|\Phi_\alpha|^2 + |\Phi_\beta|^2) + c_{4d}\sum_{\alpha\neq\beta}|C_{\alpha\beta d}|^2(|\Phi_\alpha|^2 + |\Phi_\beta|^2)\end{aligned} \tag{S24}$$

where (using $\nu \in \{c, d\}$ and $\bar{\nu} \in \{d, c\}$)

$$\begin{aligned} c_\Phi &= \sum_n \int \mathcal{G}^2_{ci}(i\omega_n, \boldsymbol{q})\mathcal{G}^2_{di}(i\omega_n, \boldsymbol{q}), \quad c_{1\nu} = \sum_n \int \mathcal{G}^2_{\nu i}(i\omega_n, \boldsymbol{q})\mathcal{G}^2_{\nu j}(i\omega_n, \boldsymbol{q}), \quad c_{2\nu} = \sum_n \int \mathcal{G}_{\nu 1}(i\omega_n, \boldsymbol{q})\mathcal{G}_{\nu 2}(i\omega_n, \boldsymbol{q})\mathcal{G}^2_{\nu 3}(i\omega_n, \boldsymbol{q}), \\ c_3 &= \sum_n \int \mathcal{G}_{d1}(i\omega_n, \boldsymbol{q})\mathcal{G}_{d2}(i\omega_n, \boldsymbol{q})\mathcal{G}_{c1}(i\omega_n, \boldsymbol{q})\mathcal{G}_{c2}(i\omega_n, \boldsymbol{q}), \quad c_{4\nu} = \sum_n \int \mathcal{G}_{\bar{\nu} i}(i\omega_n, \boldsymbol{q})\mathcal{G}_{\nu j}(i\omega_n, \boldsymbol{q})\mathcal{G}^2_{\nu i}(i\omega_n, \boldsymbol{q}). \end{aligned} \tag{S25}$$

The coefficients $c_{i\nu}$ are evaluated in Figure S5(c).

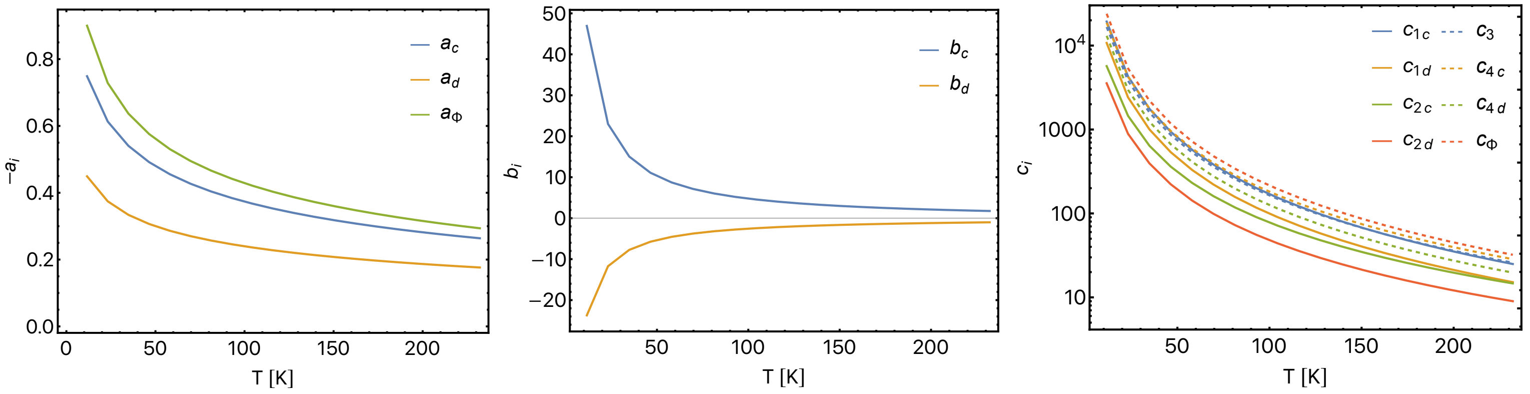

FIG. S5: Coefficients of the free energy expansion (S16) – used explicitly to obtain the phase diagram of Fig. 4 [of the main text]. Everywhere we take the infrared cut-off as temperature T. And have taken $\kappa = 2$.

VI. HONEYCOMB-KAGOME BILAYER TOY MODEL

An illustrative model of a TvHS is the following toy lattice model comprising of a honeycomb-kagome bilayer, with interlayer coupling a site on the honeycomb lattice to the the three nearest sites on the kagome lattice:

$$\begin{aligned} H_{\text{bilayer}} &= H_{\text{H}} + H_{\text{K}} + H_{\text{T}}, \\ H_{\text{H}} &= -\sum_i \left\{ t_+ \left(a_i^\dagger b_i + b_i^\dagger a_i \right) + \gamma_0 \left(a_i^\dagger a_i + b_i^\dagger b_i \right) \right\}, \\ H_{\text{K}} &= -t_- \sum_{\langle i,j \rangle} \left\{ A_i^\dagger B_j + A_i^\dagger C_j + B_i^\dagger C_j \right\} + \text{h.c.}, \\ H_{\text{T}} &= \gamma_1 \sum_i (a_i^\dagger + b_i^\dagger)(A_i + B_i + C_j) + \text{h.c.} \end{aligned} \tag{S26}$$

Here $a_i^\dagger, b_i^\dagger$ create electrons on the (a, b) sublattices of the honeycomb layer at site i, $A_i^\dagger, B_i^\dagger, C_i^\dagger$ create electrons on the kagome (A, B, C) sublattices at site i, and the sum over $\langle i, j \rangle$ enumerates nearest neighbours. The interlayer coupling γ_1 connects a given sublattice site of the honeycomb lattice to the nearest three sublattice sites of the kagome lattice, and γ_0 is an onsite energy shift which acts to increase the chemical potential in one layer relative to the other, aligning the valence and conduction bands associated to each layer. A schematic of the lattice geometry is depicted in Fig S6(a). Taking $t_+ = t_- = 1, \gamma_0 = 3, \gamma_1 = 0.25$, the bandstructure and Fermi surfaces are shown in Fig. S6(b) & (c).

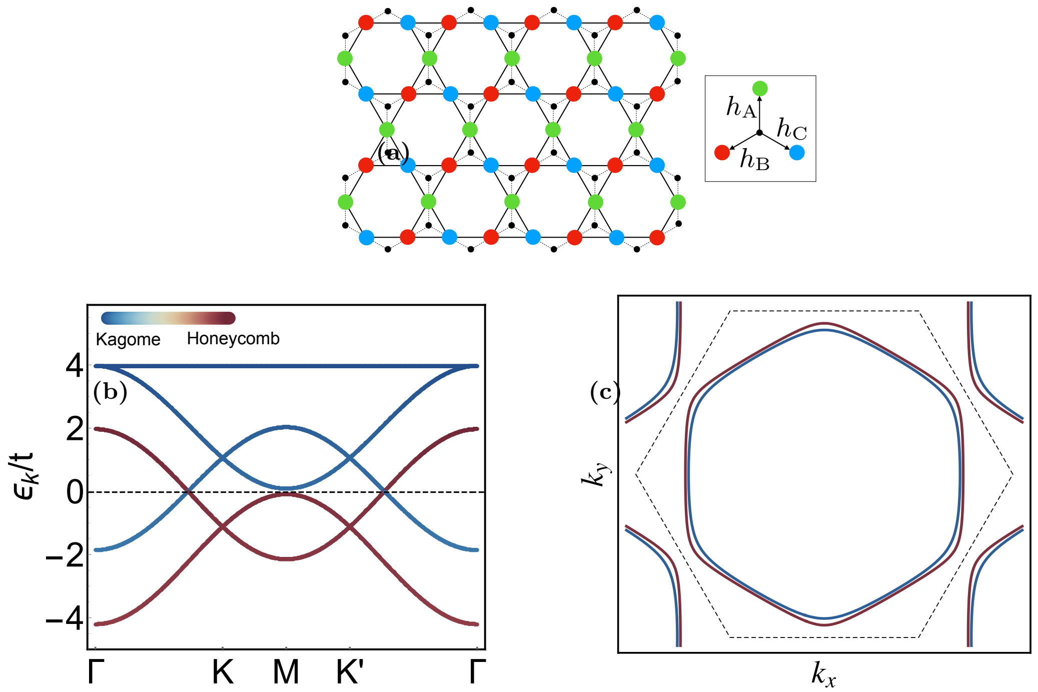

FIG. S6: **Bilayer tight binding toy model:** (a) Lattice geometry; black dots indicate the honeycomb sites connected by dashed lines, coloured dots indicate kagome sites coloured by sublattice. Inset shows nearest neighbour tunneling vectors $\boldsymbol{h}_\sigma$ connecting honeycomb and kagome sites. (b) Bandstructure and (c) Fermi surface for $\gamma_0 = 3, \gamma_1 = 0.25$. The colour scaling indicates the wavefunction weight coming from either the kagome or the honeycomb lattice. The chemical potential is shown as the dotted black line in (b), and is chosen to correspond to doping near the $\boldsymbol{M}$-point. Corresponding Fermi surface is plotted in (c), with the Brillouin zone boundary shown in dashed black.

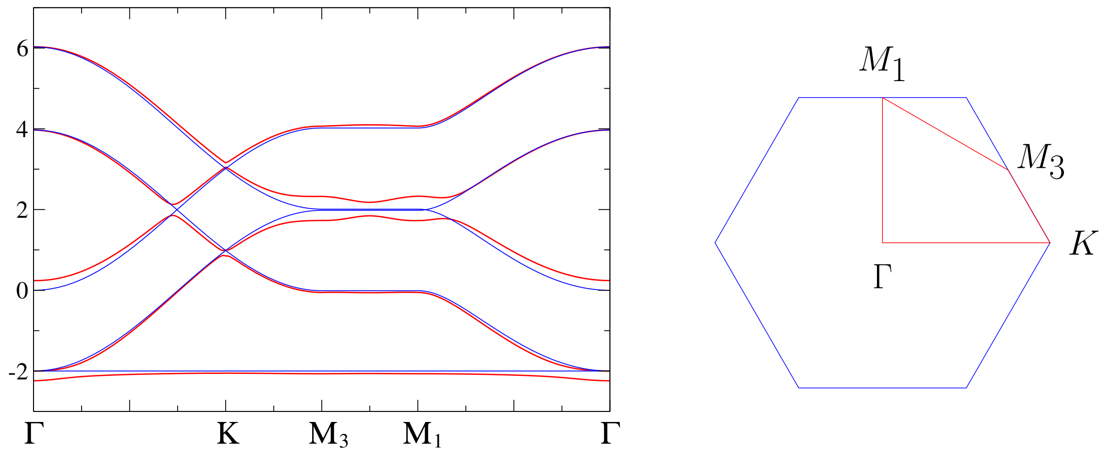

FIG. S7: **Exact diagonalisation results:** Left: the bulk dispersion for the honeycomb/kagome bilayer with parameters $t_+ = t_- = t$, $t_\perp = 0.1t$, and $\Delta = 0.3t$ (red), $\Delta = 0$ (blue). Right: the path in the Brillouin zone along which the dispersion is plotted.

VII. PROPERTIES OF THE CHIRAL EXCITONIC CONDENSATE

In this section we elaborate on the properties of the chiral excitonic condensate. The excitonic order parameter winds by a phase $\pm 4\pi$ around the Fermi surface and fully gaps the bulk dispersion (c.f. Fig S7). As a result, the mean-field Hamiltonian for the excitonic condensate describes a Chern insulator with Chern number $C = \pm 2$. In order to demonstrate the non-trivial topology, we diagonalise a mean-field Hamiltonian defined on a lattice, which provides a completion of the low-energy description we have so far considered to the entire Brillouin zone. Following on from the toy model of Section VI, we shall consider a honeycomb/kagome bilayer in an infinite ribbon geometry with zigzag edges. In addition, we shall present results for a honeycomb-honeycomb bilayers, and two-orbital kagome systems.

A. Honeycomb-kagome bilayer

In this section, we shall switch to more compact notation compared to the main text and VI, for ease of describing the real space structure of the excitonic order parameter. We introduce the simplified lattice model

$$
\begin{aligned}
H &= H_+ + H_- + H_\perp + H_\Delta \quad , \\
H_+ &= -t_+ \sum_{\langle \boldsymbol{r},\boldsymbol{r}'\rangle \in +} c^\dagger_{\boldsymbol{r}'} c_{\boldsymbol{r}} + \gamma_0 \sum_{\boldsymbol{r}\in +} c^\dagger_{\boldsymbol{r}} c_{\boldsymbol{r}} \quad , \\
H_- &= -t_- \sum_{\langle \boldsymbol{r},\boldsymbol{r}'\rangle \in \nu} c^\dagger_{\boldsymbol{r}'} c_{\boldsymbol{r}} \quad , \\
H_\perp &= \gamma_1 \sum_{\substack{\boldsymbol{r}\in\sigma=\{a,b\} \\ j=\{A,B,C\}}} c^\dagger_{\boldsymbol{r}+\boldsymbol{h}_{j\sigma}} c_{\boldsymbol{r}} + \text{h.c.} \quad , \\
H_\Delta &= \sum_{\boldsymbol{r}'\in +, \boldsymbol{r}\in -} \Delta(\boldsymbol{r}', \boldsymbol{r}) c^\dagger_{\boldsymbol{r}'} c_{\boldsymbol{r}}
\end{aligned}
\tag{S27}
$$

consisting of a honeycomb layer stacked on top of a kagome layer, where $\nu = +, -$ denote the honeycomb and kagome layers, a, b denote the sublattices of the honeycomb layer, A, B, C denote the sublattices of the kagome layer, and $\boldsymbol{h}_{j\sigma}$ are vectors connecting a site in sublattice σ in the honeycomb layer to its nearest neighbors in sublattice j in the kagome layer. The Hamiltonian consists of the bilayer toy model – with nearest neighbor hopping in the honeycomb and kagome planes with hopping energies $t_\pm$, a relative chemical potential γ_0 between the two layers, as well as tunneling γ_1 – along with an excitonic pairing term $\Delta(\boldsymbol{r}', \boldsymbol{r})$ from a site in the honeycomb layer to its three nearest

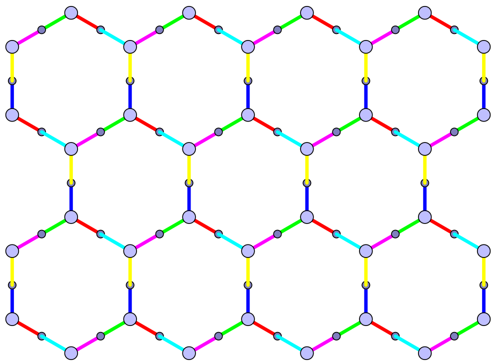

FIG. S8: The real space pairing Hamiltonian (S31) for a $d + id$ excitonic insulator ($\ell = 2$) . The large (small) circles indicate sites in the honeycomb (kagome) layer, while the coloured bonds represent the phases of the excitonic pairing; blue, green, red, yellow, magenta, and cyan bonds corresponding to the phases $e^{-\frac{i\pi}{6}}, e^{-\frac{5\pi i}{6}}, e^{i\pi}, e^{\frac{5i\pi}{6}}, e^{\frac{i\pi}{6}}, 1$.

neighbors in the kagome layer. We choose the excitonic pairing function $\Delta(\boldsymbol{r}, \boldsymbol{r'})$ so that the lattice theory possesses an equivalent continuum limit to our field theory description of the three patches surrounding the M points. The spatial wavefunctions of the eigenstates $|\boldsymbol{k} \approx \boldsymbol{M}_j, \nu\rangle$ of $H_\parallel$ in the upper and lower layers ($\nu = +, -$) are given explicitly by

$$\psi^\dagger_{\boldsymbol{k},\nu} = \frac{1}{\sqrt{N}} \sum_{\boldsymbol{r}\in\nu} \varphi_{\boldsymbol{k},\nu}(\boldsymbol{r}) c^\dagger_{\boldsymbol{r}} \tag{S28}$$

where

$$\varphi_{\boldsymbol{k},+}(\boldsymbol{r}) = \tfrac{1}{\sqrt{2}} e^{i\boldsymbol{M}_j\cdot\boldsymbol{r}}, \quad \varphi_{\boldsymbol{k},-}(\boldsymbol{r}) = \begin{cases} \frac{1}{\sqrt{2}} e^{i\boldsymbol{M}_j\cdot\boldsymbol{r}} & j \neq \sigma_j \\ 0 & j = \sigma_j \end{cases} \tag{S29}$$

where $\boldsymbol{k} \approx \boldsymbol{M}_j$, and the sublattice index $(\sigma_1, \sigma_2, \sigma_3) = (a, b, c)$ corresponds to the sites in the kagome lattice for which the 2D projection of the bond vector $\boldsymbol{h}_{\sigma_j A}$ is parallel to $\boldsymbol{M}_j$. Near the M points, the effective Hamiltonian projected onto states near the Fermi surface is given by

$$\begin{aligned} H_\Delta &= \sum_{j,\boldsymbol{k}\approx\boldsymbol{M}_j} \Delta_j \psi^\dagger_{\boldsymbol{k},+}\psi_{\boldsymbol{k},-} + \text{h.c.}, \\ \Delta_j &= \tfrac{1}{2} e^{\frac{i\pi}{6}} \sum_{\sigma\neq j} \Delta(\boldsymbol{r}_A + \boldsymbol{h}_{\sigma A}, \boldsymbol{r}_A) - \Delta(\boldsymbol{r}_B + \boldsymbol{h}_{\sigma B}, \boldsymbol{r}_B) \end{aligned} \tag{S30}$$

where $\boldsymbol{r}_A, \boldsymbol{r}_B$ are the coordinates of the A and B sites in the first unit cell. The direct tunneling Hamiltonian $H_\perp$ does not appear in the effective Hamiltonian near the M points, since the contributions from hopping processes involving opposite sublattices in the honeycomb layer interfere destructively. A $d \pm id$ order parameter $(\Delta_1, \Delta_2, \Delta_3) = (\Delta, \Delta e^{\frac{2\pi i}{3}\ell}, \Delta e^{\frac{4\pi i}{3}\ell})$ for $\ell = \pm 2$ implies that the real-space pairing functions are given by

$$\begin{aligned} \Delta(\boldsymbol{r}_A + \boldsymbol{h}_{aA}, \boldsymbol{r}_A) &= -\Delta(\boldsymbol{r}_B + \boldsymbol{h}_{aB}, \boldsymbol{r}_A) = e^{-\frac{i\pi}{6}}\Delta \\ \Delta(\boldsymbol{r}_A + \boldsymbol{h}_{bA}, \boldsymbol{r}_A) &= -\Delta(\boldsymbol{r}_B + \boldsymbol{h}_{bB}, \boldsymbol{r}_A) = e^{-\frac{i\pi}{6}+\frac{2\pi i}{3}\ell}\Delta \\ \Delta(\boldsymbol{r}_A + \boldsymbol{h}_{cA}, \boldsymbol{r}_A) &= -\Delta(\boldsymbol{r}_B + \boldsymbol{h}_{cB}, \boldsymbol{r}_A) = e^{-\frac{i\pi}{6}+\frac{4\pi i}{3}\ell}\Delta \end{aligned} \tag{S31}$$

which we illustrate in Fig. S8.

We diagonalise the mean-field lattice Hamiltonian for an infinite ribbon geometry in the $\ell = 2$ phase with zigzag edges for parameters $t_+ = t_-$, $\gamma = 0.1t$, $\Delta = 0.4t$, and show the 1D dispersion as a function of momentum k_x along the ribbon in Fig. S9 in the main text, with

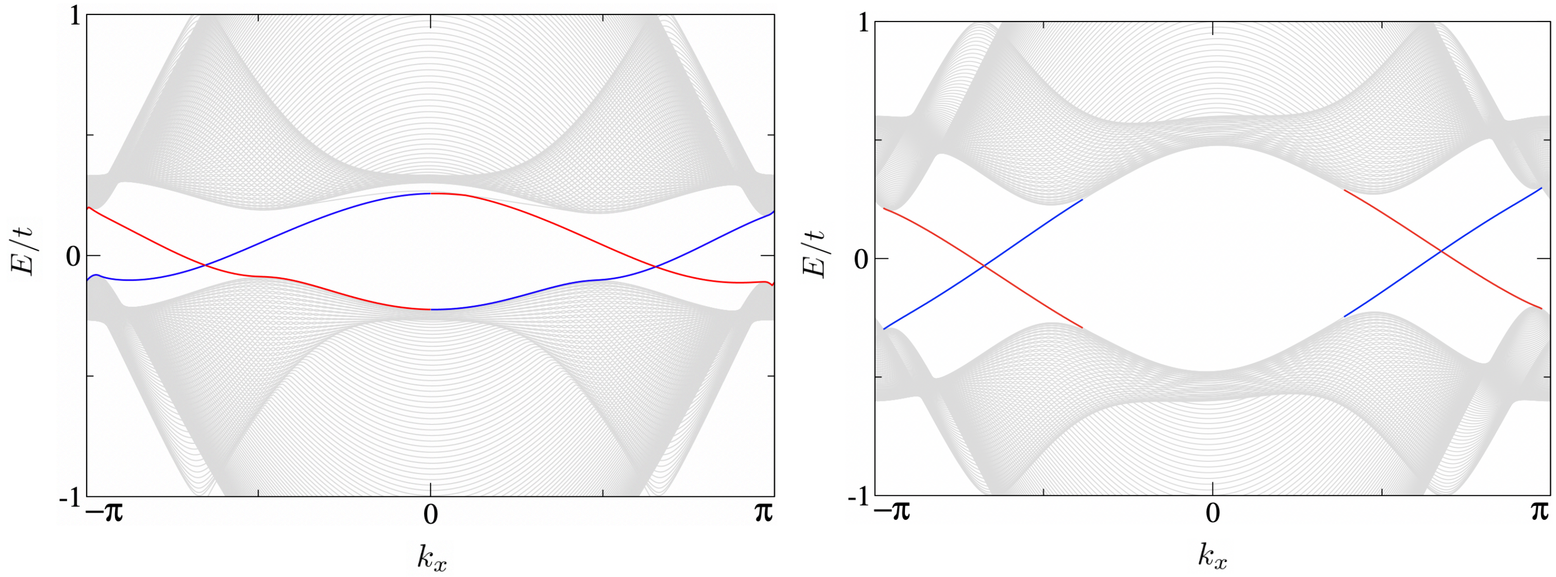

FIG. S9: Right: The 1D dispersion of an AA stacked honeycomb bilayer for an infinite ribbon with width 120 unit cells, with parameters $t_+ = t_- = t$, $t_\perp = 0.1t$, $\Delta = 0.3t$. The edge states on the top/bottom of the ribbon are plotted in red/blue. Left: kagome

energy in units of t_+. The dispersion exhibits two chiral edge modes, with the left-(right-)movers propagating along the top (bottom) of the ribbon represented by red (blue). The bulk dispersion is shown in Fig. S7. For comparison, we have also diagonalised a lattice model consisting of two AA stacked honeycomb layers. In this case the chiral d-wave order generates pairing between an A site in one layer and its nearest neighboring B sites in the opposite layer, with the pairing function

$$\begin{aligned}\Delta(\boldsymbol{r}_A + \boldsymbol{d}_1 + \boldsymbol{c}, \boldsymbol{r}_A) &= -\Delta(\boldsymbol{r}_B - \boldsymbol{d}_1 + \boldsymbol{c}, \boldsymbol{r}_B) = \tfrac{1}{2}\Delta \\ \Delta(\boldsymbol{r}_A + \boldsymbol{d}_1 + \boldsymbol{c}, \boldsymbol{r}_A) &= -\Delta(\boldsymbol{r}_B - \boldsymbol{d}_1 - \boldsymbol{c}, \boldsymbol{r}_B) = \tfrac{1}{2}e^{\frac{2\pi i}{3}\ell}\Delta \\ \Delta(\boldsymbol{r}_A + \boldsymbol{d}_1 - \boldsymbol{c}, \boldsymbol{r}_A) &= -\Delta(\boldsymbol{r}_B - \boldsymbol{d}_1 - \boldsymbol{c}, \boldsymbol{r}_B) = \tfrac{1}{2}e^{\frac{4\pi i}{3}\ell}\Delta \end{aligned} \tag{S32}$$

The 1D dispersion for an infinite ribbon in the $\ell = 2$ phase with parameters $t_+ = t_- = t, \gamma_0 = 2t, \gamma_1 = 0.1t, \Delta = 0.3t$ is shown in Fig. S9, with $t_\pm, \gamma_0, \gamma_1$ referring (as in the kagome model) to the intralayer hopping energies, chemical potential shift between the layers and vertical hopping energies respectively.

B. Two-orbital kagome systems

We now derive the real space description for two-orbital kagome systems – arising due to a kagome-kagome bilayer or pair of orbitals in a monolayer – and diagonalise the excitonic mean field Hamiltonian for this case as well. First, we consider a kagome lattice with two orbitals $\nu = \pm$ described by the Hamiltonian

$$H = -t \sum_{\langle \boldsymbol{r}', \boldsymbol{r} \rangle} c_\nu^\dagger(\boldsymbol{r}') c_\nu(\boldsymbol{r}) - \frac{1}{2} \sum_{\boldsymbol{r}} \nu \mu_\nu c_\nu^\dagger(\boldsymbol{r}) c_\nu(\boldsymbol{r}) \tag{S33}$$

where μ_ν is a relative energy shift. We go to the momentum representation

$$c^\dagger_{\boldsymbol{k},\sigma,\nu} = \tfrac{1}{\sqrt{N}} \sum_{\boldsymbol{r} \in \sigma} e^{i\boldsymbol{k}\cdot\boldsymbol{r}} c_\nu^\dagger(\boldsymbol{r}) \tag{S34}$$

which gives us

$$H(\boldsymbol{k}) = \sum_{\boldsymbol{k},\sigma,\sigma',\nu} c^\dagger_{\boldsymbol{k},\sigma',\nu} \mathcal{H}_{\sigma'\sigma;\nu}(\boldsymbol{k}) c_{\boldsymbol{k},\sigma,\nu}$$

$$\mathcal{H}_{\sigma'\sigma;\nu} = -\tfrac{\nu\mu_\nu}{2}\delta_{\sigma\sigma'} - 2t \begin{pmatrix} 0 & \cos(\boldsymbol{k}\cdot\boldsymbol{d}_c) & \cos(\boldsymbol{k}\cdot\boldsymbol{d}_b) \\ \cos(\boldsymbol{k}\cdot\boldsymbol{d}_c) & 0 & \cos(\boldsymbol{k}\cdot\boldsymbol{d}_a) \\ \cos(\boldsymbol{k}\cdot\boldsymbol{d}_b) & \cos(\boldsymbol{k}\cdot\boldsymbol{d}_a) & 0 \end{pmatrix}_{\sigma'\sigma} \tag{S35}$$

where $\boldsymbol{d}_a, \boldsymbol{d}_b, \boldsymbol{d}_c = \boldsymbol{d}_j$ are 2D vectors satisfying $\boldsymbol{d}^+_j = \frac{a}{2} e^{\frac{2\pi i}{3}j}$ with $\{a,b,c\}$ corresponding to $j = \{0,1,2\}$. At the M points $\boldsymbol{M}_j$, satisfying $\boldsymbol{M}^+_j = \frac{2\pi}{\sqrt{3}a} i e^{\frac{2\pi i}{3}j}$, we have

$$\boldsymbol{M}_j \cdot \boldsymbol{d}_i = \mathrm{Re}\,\boldsymbol{M}^+_j \boldsymbol{d}^-_i = \begin{cases} 0 \;, & j = i \\ \mp\frac{\pi}{2} \;, & j = i \pm 1 \mod 3 \end{cases} \tag{S36}$$

At the $\boldsymbol{M}_1$ point we have

$$\mathcal{H}_\nu(\boldsymbol{k} \approx \boldsymbol{M}_j) = -\tfrac{\nu\mu_\nu}{2} - 2t \begin{pmatrix} 0 & 0 & 0 \\ 0 & 0 & 1 \\ 0 & 1 & 0 \end{pmatrix} \tag{S37}$$

which has a spectrum $E = \{-\frac{\nu\mu_\nu}{2} - 2t, -\frac{\nu\mu_\nu}{2}, -\frac{\nu\mu_\nu}{2} + 2t\}$ with corresponding creation operators

$$\psi^\dagger_{\boldsymbol{k},m,\nu} = \tfrac{1}{\sqrt{2}}(b^\dagger_{\boldsymbol{k},\nu} + c^\dagger_{\boldsymbol{k},\nu}) \;\;,\;\; \psi^\dagger_{\boldsymbol{k},p,\nu} = a^\dagger_{\boldsymbol{k},\nu} \;\;,\;\; \psi^\dagger_{\boldsymbol{k},m',\nu} = \tfrac{1}{\sqrt{2}}(b^\dagger_{\boldsymbol{k},\nu} - c^\dagger_{\boldsymbol{k},\nu}) \;\;. \tag{S38}$$

Note that at the Γ point, we have

$$\mathcal{H}_\nu(\boldsymbol{k} = \Gamma) = -\tfrac{\nu\mu_\nu}{2} - 2t \begin{pmatrix} 0 & 1 & 1 \\ 1 & 0 & 1 \\ 1 & 1 & 0 \end{pmatrix} \tag{S39}$$

which has eigenvalues $E = \{-\frac{\nu\mu_\nu}{2} - 4t, -\frac{\nu\mu_\nu}{2} + 2t, -\frac{\nu\mu_\nu}{2} + 2t\}$. This allows us to identify the highest band, with energy $E = -\frac{\nu\mu_\nu}{2} + 2t$, as the flat band, and the lower two bands as those hosting Dirac points. We consider the case where the middle band of the $\nu = +$ orbital is aligned with the lower band of the $\nu = +$ orbital, so $\frac{\mu_\nu}{2} - 2t = -\frac{\mu_\nu}{2} \to \mu_\nu = 2t$. Let us now consider an additional excitonic pairing term

$$\begin{aligned} H_{\mathrm{ex}} &= \sum_{\boldsymbol{r},\boldsymbol{d}_i,\pm} \Delta(\boldsymbol{r} \pm \boldsymbol{d}_i, \boldsymbol{r}) c^\dagger_-(\boldsymbol{r} \pm \boldsymbol{d}_i) c_+(\boldsymbol{r}) + \mathrm{H.c.} \\ &= \sum_{\boldsymbol{k},\sigma,\sigma'} \sum_{\boldsymbol{r}_\sigma \pm \boldsymbol{d}_i \in \sigma'} \Delta(\boldsymbol{r}_\sigma \pm \boldsymbol{d}_i, \boldsymbol{r}_\sigma) e^{\mp i \boldsymbol{k}\cdot\boldsymbol{d}_i} c^\dagger_{\boldsymbol{k},\sigma',-} c_{\boldsymbol{k},\sigma,+} + \mathrm{H.c.} \end{aligned} \tag{S40}$$

We project onto the bands that touch at the M points, i.e. $\psi^\dagger_{\boldsymbol{k},\nu=+} = \psi^\dagger_{\boldsymbol{k},p,+}$ and $\psi^\dagger_{\boldsymbol{k},-} = \psi^\dagger_{\boldsymbol{k},m,-}$, via the relations

$$\begin{aligned} c^\dagger_{\boldsymbol{k}\approx\boldsymbol{M}_j,\sigma=j,\nu} &= \psi^\dagger_{\boldsymbol{k}\approx\boldsymbol{M}_j,p,\nu} \;\;, \\ c^\dagger_{\boldsymbol{k}\approx\boldsymbol{M}_j,\sigma=j\pm1,\nu} &= \tfrac{1}{\sqrt{2}}(\psi^\dagger_{\boldsymbol{k},m,\nu} \pm \psi^\dagger_{\boldsymbol{k},m',\nu}) \end{aligned} \tag{S41}$$

where the relation $\sigma = j \pm 1$ is understood to hold mod 3. We then obtain

$$\begin{aligned} H_{\mathrm{ex}} &= \sum_j \sum_{\boldsymbol{k}\approx\boldsymbol{M}_j,\sigma=j,\sigma'\neq j} \sum_{\boldsymbol{r}_\sigma \pm \boldsymbol{d}_i \in \sigma'} \Delta(\boldsymbol{r}_\sigma \pm \boldsymbol{d}_i, \boldsymbol{r}_\sigma) e^{\mp i \boldsymbol{M}_j\cdot\boldsymbol{d}_i} c^\dagger_{\boldsymbol{k},\sigma',-} c_{\boldsymbol{k},\sigma,+} + \mathrm{H.c.} \\ &= \tfrac{1}{\sqrt{2}} \sum_j \sum_{\boldsymbol{k}\approx\boldsymbol{M}_j} \sum_{i\neq j} \Delta(\boldsymbol{r}_j \pm \boldsymbol{d}_i, \boldsymbol{r}_j) e^{\mp i \boldsymbol{M}_j\cdot\boldsymbol{d}_i} \psi^\dagger_{\boldsymbol{k},-} \psi_{\boldsymbol{k},+} \equiv \sum_j \sum_{\boldsymbol{k}\approx\boldsymbol{M}_j} \Delta_j \psi^\dagger_{\boldsymbol{k},-} \psi_{\boldsymbol{k},+} \end{aligned} \tag{S42}$$

with

$$\Delta_j = \frac{1}{\sqrt{2}} \sum_{i \neq j} \Delta(\boldsymbol{r}_j \pm \boldsymbol{d}_i, \boldsymbol{r}_j) e^{\mp i \boldsymbol{M}_j \cdot \boldsymbol{d}_i}$$

$$= \frac{1}{\sqrt{2}} (\Delta(\boldsymbol{r}_j + \boldsymbol{d}_{j+1}, \boldsymbol{r}_j) e^{-i\boldsymbol{M}_j \cdot \boldsymbol{d}_{j+1}} + \Delta(\boldsymbol{r}_j - \boldsymbol{d}_{j+1}, \boldsymbol{r}_j) e^{i\boldsymbol{M}_j \cdot \boldsymbol{d}_{j+1}} + \Delta(\boldsymbol{r}_j + \boldsymbol{d}_{j-1}, \boldsymbol{r}_j) e^{-i\boldsymbol{M}_j \cdot \boldsymbol{d}_{j-1}} + \Delta(\boldsymbol{r}_j - \boldsymbol{d}_{j-1}, \boldsymbol{r}_j) e^{i\boldsymbol{M}_j \cdot \boldsymbol{d}_{j-1}})$$

$$= \frac{1}{\sqrt{2}} (-i\Delta(\boldsymbol{r}_j + \boldsymbol{d}_{j+1}, \boldsymbol{r}_j) + i\Delta(\boldsymbol{r}_j - \boldsymbol{d}_{j+1}, \boldsymbol{r}_j) + i\Delta(\boldsymbol{r}_j + \boldsymbol{d}_{j-1}, \boldsymbol{r}_j) - i\Delta(\boldsymbol{r}_j - \boldsymbol{d}_{j-1}, \boldsymbol{r}_j)) \tag{S43}$$

There is freedom in the choice of $\Delta(\boldsymbol{r} + \boldsymbol{d}_i, \boldsymbol{r}_j)$, however note that setting it to a constant value results in zero. One possibility is

$$\Delta(\boldsymbol{r}_j \pm \boldsymbol{d}_{j'}, \boldsymbol{r}_j) = \pm \frac{1}{\sqrt{6}} \Delta_j e^{\frac{2\pi i}{3}(j'-j)} \quad , \tag{S44}$$

since we have

$$\frac{1}{\sqrt{2}} (-i\Delta(\boldsymbol{r}_j + \boldsymbol{d}_{j+1}, \boldsymbol{r}_j) + i\Delta(\boldsymbol{r}_j - \boldsymbol{d}_{j+1}, \boldsymbol{r}_j) + i\Delta(\boldsymbol{r}_j + \boldsymbol{d}_{j-1}, \boldsymbol{r}_j) - i\Delta(\boldsymbol{r}_j - \boldsymbol{d}_{j-1}, \boldsymbol{r}_j))$$

$$= -\frac{i}{\sqrt{12}} \Delta_j e^{-\frac{2\pi i}{3} j} \left[2e^{\frac{2\pi i}{3}(j+1)} - 2e^{\frac{2\pi i}{3}(j-1)} \right] = \Delta_j \tag{S45}$$

Thus for $\Delta_j = \Delta_0 e^{\frac{2\pi i}{3} \ell j}$ we have

$$\Delta(\boldsymbol{r}_j \pm \boldsymbol{d}_{j'}, \boldsymbol{r}_j) = \pm \frac{1}{\sqrt{6}} \Delta_0 e^{\frac{2\pi i}{3}(j' + (\ell - 1)j)} \tag{S46}$$

The resulting real space Hamiltonian is diagonalised for a ribbon geometry with 60 unit cells, the result being Fig. 4 of the main text.

[1] X. Wu et al., "Nature of unconventional pairing in the kagome superconductors AV_3Sb_5", Phys. Rev. Lett. **127**, 177001 (2021).

[2] T. Park, M. Ye and L. Balents "Electronic instabilities of kagome metals: saddle points and Landau theory", Phys. Rev. B **104**, 035142 (2021).

[3] S. Maiti and A. V. Chubukov, "Superconductivity from repulsive interaction", AIP Conference Proceedings **1550**, 3 (2013).
